# A Very Short Uranium(IV)–Rhodium(I) Bond with Net Double‐Dative Bonding Character

**DOI:** 10.1002/anie.201803493

**Published:** 2018-04-27

**Authors:** Erli Lu, Ashley J. Wooles, Matthew Gregson, Philip J. Cobb, Stephen T. Liddle

**Affiliations:** ^1^ School of Chemistry The University of Manchester Oxford Road Manchester M13 9PL UK

**Keywords:** carbenes, C−H activation, metal–metal bonding, rhodium, uranium

## Abstract

Reaction of [U{C(SiMe_3_)(PPh_2_)}(BIPM)(μ‐Cl)Li(TMEDA)(μ‐TMEDA)_0.5_]_2_ (BIPM=C(PPh_2_NSiMe_3_)_2_; TMEDA=Me_2_NCH_2_CH_2_NMe_2_) with [Rh(μ‐Cl)(COD)]_2_ (COD=cyclooctadiene) affords the heterotrimetallic U^IV^−Rh^I^_2_ complex [U(Cl)_2_{C(PPh_2_NSiMe_3_)(PPh[C_6_H_4_]NSiMe_3_)}{Rh(COD)}{Rh(CH(SiMe_3_)(PPh_2_)}]. This complex has a very short uranium–rhodium distance, the shortest uranium–rhodium bond on record and the shortest actinide–transition metal bond in terms of formal shortness ratio. Quantum‐chemical calculations reveal a remarkable RhI→→ U^IV^ net double dative bond interaction, involving Rh^I^ 4d${}_{z{}^{2}}$ ‐ and 4d_xy/xz_‐type donation into vacant U^IV^ 5f orbitals, resulting in a Wiberg/Nalewajski–Mrozek U−Rh bond order of 1.30/1.44, respectively. Despite being, formally, purely dative, the uranium–rhodium bonding interaction is the most substantial actinide–metal multiple bond yet prepared under conventional experimental conditions, as confirmed by structural, magnetic, and computational analyses.

The study of metal–metal bonds is an enduring, fascinating, and burgeoning field of endeavour that impacts many areas of molecular and materials chemistry.^1^ Although metal–metal bonding is prevalent across most of the Periodic Table,^2^ that of f‐block‐metal complexes is, relatively speaking, still in its infancy.^3^ Under conventional experimental conditions, apart from a few but notable lanthanide–metal bonds,^4^ the number of molecular actinide–metal bonds has grown, but only slowly.^3^, ^5^, ^6^ Studying uranium–metal bonds is of particular interest because there is continued debate over the level and nature of covalency in uranium–ligand bonding.^7^ Also, although uranium–E (E=main‐group element) bonding has been intensively investigated in recent years,^8^ U−M complexes (M=transition‐metal) remain scarce,^3^, ^5^ but such complexes present opportunities to probe novel bonding motifs that may arise from the electronegativity mismatch of pairing uranium with a transition metal.

The majority of uranium–metal complexes contain a polarised covalent σ‐bond, which may be augmented by a weak π‐bonding interaction.3b, 5 However, examples with dative metal donors are few, since they lack a substantial electrostatic attractive force to pair the two metal ions. Notable examples include U−Al/−Ga complexes utilising [AlCp*] and [Ga(Cp*)],5n,5p a trio of Group 10 derivatives [(I)UM(μ‐OPAr)_3_] (M=Ni, Pd, Pt; OPAr=C_6_H_2_‐1‐PPh_2_‐2‐O‐3‐Bu^t^‐5‐Me),5c and two Group 9 derivatives [U(I)(μ‐I)Rh(μ‐OPAr)_3_] and [U(I)_2_(μ‐OPAr)_2_Rh(μ‐I)]_2_,5a though in all cases these systems seem to be limited to polar single U−M bonds. Very recently, [UFe(CO)_3_]^−^ and [OUFe(CO)_3_]^−^ with covalent‐σ and double‐dative‐π bonds, were reported in the gas phase,^9^ which hints that novel U−M bonds might await discovery under normal experimental conditions. When characterising metal–metal bonds, bond order is an intuitive and important metric to consider, but perhaps the best benchmark is the formal shortness ratio (FSR_MM′_=MM′ distance/sum of MM′ covalent radii),^1^ because this enables comparisons to be drawn about the relative shortness of a metal–metal bond by normalising different metal covalent radii. Using Pyykkö’s values,^10^ the current state‐of‐the‐art for actinide–metal derivatives prepared under normal conditions is a FSR_UNi_ value of 0.90 for [(I)UNi(μ‐OPAr)_3_],5c whereas complexes with U−Re,5i,5k,5m U−Ru,5g U−Rh,5a and U−Co5e,5f bonds have average FSR_UM_ values of 0.98, 1.01, 0.93, and 1.04, respectively. These FSR values are higher than often observed in stronger M−M′ bonding, where values less than 0.8 can be found for high M−M′ bond order species,^11^ and this highlights the knowledge gap in the field in terms of strongly and multiply bonded intermetallic bonds.

Herein, we report the synthesis of a heterotrimetallic uranium(IV)–rhodium(I)–rhodium(I) complex that contains a very short uranium–rhodium bond. This is the shortest uranium–rhodium bond distance on record, and under conventional experimental conditions is the shortest actinide–metal bond yet prepared according to the FSR criterion. Quantum‐chemical calculations surprisingly reveal a RhI→→ U^IV^ double dative bond interaction with the highest actinide–metal bond order to date outside of matrix isolation or gas‐phase species.

Initially on a small scale, under an N_2_ atmosphere at room temperature, the uranium silyl–phosphino–carbene complex [U{C(SiMe_3_)(PPh_2_)}(BIPM)(μ‐Cl)Li(TMEDA)(μ‐TMEDA)_0.5_]_2_ (**1**, BIPM=C(PPh_2_NSiMe_3_)_2_; TMEDA=Me_2_NCH_2_CH_2_NMe_2_)^12^ was treated with the rhodium chloride compound [Rh(μ‐Cl)(COD)]_2_ (COD=cyclooctadiene) in D_6_‐benzene (Scheme 1).^13^ This led to the otherwise insoluble **1** dissolving, and the red solution turning black. After standing for three days, NMR spectroscopy of this mixture revealed that one major uranium‐containing product was formed along with free COD, TMEDA, and some minor byproducts. The product is formulated as the heterotrimetallic U^IV^−Rh^I^_2_ complex [U(Cl)_2_{C(PPh_2_NSiMe_3_)(PPh[C_6_H_4_]NSiMe_3_)}{Rh(COD)}{Rh(CH(SiMe_3_)(PPh_2_)}] (**2**). The ^31^P NMR spectrum of **2** consists of three resonances at 17.7, −356.1, and −533.2 ppm; the former is a doublet with a characteristic *J*_RhP_ coupling constant of 91 Hz (^103^Rh, *I*=1/2 , 100 %), whereas the latter two are characteristic of a BIPM ligand coordinated to uranium(IV) where the two phosphorus centres are magnetically inequivalent (^2^ *J*_PP_ not resolved).^12^ The ^1^H NMR spectrum is also consistent with this asymmetry, showing multiple resonances. However, this could not be resolved in detail since once **2** precipitates from solution it cannot be redissolved in non‐polar solvents and it decomposes in polar solvents. However, analyses that could be obtained support the formulation of **2**. Scaling the reaction up in toluene afforded dark red crystals of **2** in 50 % isolated yield (Scheme 1).

**Scheme 1:**
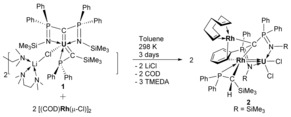
Synthesis of **2** from **1** and [Rh(μ‐Cl)(COD)]_2_. COD=cyclooctadiene, TMEDA=*N*,*N*,*N*′,*N*′‐tetramethylethylenediaine.

To confirm the formulation of **2**, its solid‐state molecular structure was determined (Figure 1).^13^ Several salient features emerge from the structure of **2**. The uranium ion adopts a trigonal bipyramidal geometry, with no bond to the BIPM methanediide centre, where Cl2 and Rh1 ions occupy the axial sites. Rh1 is further coordinated to a chelating silyl–phosphino–alkyl that derives from the corresponding carbene in **1**, an *ortho*‐carbon of a deprotonated BIPM phenyl ring that accounts well for the source of the alkyl proton, and the BIPM methanediide centre. The Rh1 ion thus adopts a square‐based pyramidal geometry. The Rh2 ion is coordinated by a COD ligand, the BIPM methanediide centre, and the same *ortho*‐phenyl carbon as Rh1. Thus, Rh2 formally adopts a square planar geometry.

**Figure 1:**
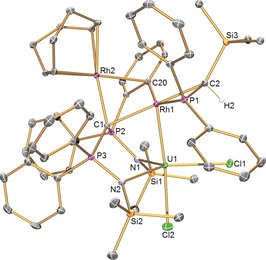
Molecular structure of **2** at 120 K.^20^ Ellipsoids set at 40 % probability; hydrogen atoms (except H2) and lattice solvent molecules are omitted for clarity. Selected bond lengths [Å]: U1−Rh1 2.5835(3), U1−Cl1 2.6481(10), U1−Cl2 2.6332(12), U1−N1 2.336(3), U1−N2 2.359(3), U1−C1 2.870(4), Rh1−C1 2.252(4), Rh1−C2 2.190(4), Rh1−P1 2.2808(10), Rh1−C20 2.081(4), Rh1⋅⋅⋅Rh2 2.8399(4), Rh2−C1 2.146(4), Rh2−C20 2.378(4).

The U1−Rh1 distance of 2.5835(3) Å is very short, being about 0.37 Å shorter than the sum of the single bond covalent radii of U and Rh (2.95 Å),^10^ and about 0.2 Å shorter than the only two other structurally authenticated U−Rh bonds in [U(I)(μ‐I)Rh(μ‐OPAr)_3_] [2.7630(5) Å] and [U(I)_2_(μ‐OPAr)_2_Rh(μ‐I)]_2_ [2.7601(5) Å].5a Interestingly, the sum of the covalent double‐bond radii of U and Rh (2.44 Å) is only about 0.14 Å shorter than the U−Rh distance in **2**.^10^ The FSR_URh_ value for **2** is 0.87, which supersedes the previous FSR_UM_ value for the U−Ni bond distance of 2.527(2) Å in [(I)UNi(μ‐OPAr)_3_]5c and FSR_URh_ values of 0.93 for [U(I)(μ‐I)Rh(μ‐OPAr)_3_] and [U(I)_2_(μ‐OPAr)_2_Rh(μ‐I)]_2_;5a it is germane to note that the U−Rh distance in **2** is only about 0.05 Å longer than the U−Ni distance above, even though the single‐bond covalent radius of Rh (1.25 Å) is 0.15 Å greater than that of Ni (1.10 Å),^10^ which implies a strong U−Rh bonding interaction in **2**.

The U⋅⋅⋅C_BIPM_ distance of 2.870(4) Å in **2** is too long to be considered as bonding, but the methanediide centre is clearly bonded to Rh1 and Rh2 with bond lengths of 2.252(4) and 2.146(4) Å, respectively. These distances are slightly longer than the sum of the single‐bond radii of Rh and C (2.0 Å),^10^ reflecting the bridging nature of C1 and that each Rh^I^ ion in **2** is coordinated to multiple anionic carbon donor ligands. The Rh1−C2 and Rh1−C20 distances of 2.190(4) and 2.081(4) Å are consistent with direct σ‐bonding to sp^3^‐alkyl and anionic sp^2^ phenyl groups,^14^ respectively, whereas the Rh2−C20 distance is longer at 2.378(4) Å reflecting the “side‐on” coordination mode of this interaction. The compressed tetrahedral geometry at C2 contrasts to the trigonal planar geometry of the silyl–phosphino–carbene in **1**,^12^ being reminiscent of other examples of this ligand in its alkyl form.^15^ Lastly, the Rh1⋅⋅⋅Rh2 distance of 2.8399(4) Å suggests there is no significant Rh−Rh bonding interaction (sum of single bond covalent radii for Rh=2.50 Å),^10^ consistent with their 4d^8^ rhodium(I) closed‐shell formulations.

At 298 K the magnetic moment of **2** is about 3.2 μ_B_ and this smoothly decreases to about 2.3 μ_B_ at about 50 K, at which point the magnetic moment decreases more sharply reaching a value of about 0.85 μ_B_ at 2 K and tending to zero, Figure 2; the latter value is about 0.5 μ_B_ higher than is usual for the temperature independent paramagnetism of uranium(IV) alone, which otherwise is a magnetic singlet at low temperature. This is characteristic behaviour of ^3^H_4_ uranium(IV) when coordinated to a strongly donating, usually multiply bonded, ligand,5f, 16 such as the silyl–phosphino–carbene ligand in **1**.^12^ The data are consistent with uranium(IV) paramagnetism alone, consistent with the closed‐shell 4d^8^ rhodium(I) formulations. The imino and chloride co‐ligands of uranium in **2** have never on their own produced unusual magnetic behaviour in uranium–BIPM derivatives,^17^ and so we conclude that the atypical magnetic behaviour of **2** results from the Rh1 centre being a strong donor ligand, which implies that it is a multiple bond donor to uranium, consistent with the short U1−Rh1 distance in **2**.

**Figure 2:**
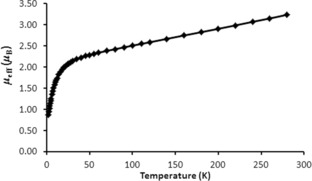
Variable temperature magnetism of **2** over the temperature range 2 to 298 K. The solid line is a guide to the eye only.

To understand the electronic structure of **2**, we performed quantum‐chemical calculations on the whole molecule of **2**. Unfortunately, geometry optimisations proved intractable; it would seem that this is due to the existence of multiple, local shallow minima on a very flat potential energy surface, since a single‐point energy calculation performed on coordinates from the crystal structure of **2** converged straightforwardly.

The α‐spin densities on U1 (−2.33), Rh1 (−0.11), and Rh2 (+0.01) are consistent with their proposed oxidation states, noting that Rh1 is coordinated to three anionic donor ligands. For all other atoms the α‐spin densities are very close to zero in all cases, indicating that the unpaired electrons in **2** are principally located at uranium, consistent with the SQUID magnetometry data. The computed MDC_q_ charges for U1 (+1.35), Rh1 (+1.48), and Rh2 (+0.52) support the notion that Rh1 is a significant donor of electron density to U1 since computed BIPM–uranium(IV) charges tend to be greater than +2 and clearly the charge on Rh1 is substantially greater than Rh2 considering their +1 oxidation states and ligand donor sets. The C1 charge of −1.57 confirms its methanediide nature.

The presence of a significant U−Rh interaction in **2** is supported by computed U1−Rh1 Wiberg bond order (WBO) and Nalewajski–Mrozek bond order (NMBO) values of 1.30 and 1.44, respectively. For comparison, this is almost twice that of the U−Ni bond in [(I)UNi(μ‐OPAr)_3_] (WBO=0.72)5c and greater than that of the U−Re bond in [U(ReCp_2_){HC(SiMe_2_NC_5_H_3_‐3,5‐Me_2_)_3_}] (NMBO=1.30).5k Since the U1−Rh1 bonding interaction in **2** is formally dative, those comparisons are significant because the U−Ni bond in the former is also dative and half the U−Rh bond order of **2**, whereas the U−Re bond is a covalent σ and dative π combination but still a smaller NMBO than the U−Rh NMBO in **2**. Irrespective of which bond order metric is used, the U1−Rh1 bond order in **2** is the highest for any U−M complex prepared under conventional experimental conditions. Indeed, it is exceeded only by the U−Fe bonds in the gas‐phase species [UFe(CO)_3_]^−^ and [OUFe(CO)_3_]^−^ where covalent‐σ double‐dative π bonds with bond orders of 1.9–3.0 were computed.^9^ The Rh1−Rh2 Wiberg bond order is computed to be 0.1, again consistent with little Rh−Rh bonding character.

As the above suggests multiple bond character between U1 and Rh1, we inspected the Kohn–Sham molecular orbitals (KSMOs) of **2**. There are three KSMOs with significant U1−Rh1 character, which are HOMO−4, −6, and −7, Figure 3. HOMO−7 mainly involves a dative σ‐donation from a predominantly 4d${}_{z{}^{2}}$ orbital of rhodium to vacant uranium 5f orbitals (Figure 3 a). For HOMO−6, the intermetallic interaction principally involves a 4d_*xz*_‐type orbital of the rhodium, but because the Rh1 fragment departs from perfect square‐based pyramidal geometry owing to steric constraints it is not optimally aligned and slips to engage in a weak quasi‐σ symmetry interaction with only one orbital lobe (Figure 3 b). HOMO−4 is less clear‐cut due to the delocalised nature of this molecular orbital, with orbital coefficients from the silyl–phosphino–alkyl intruding, but the resulting U−Rh interaction has quasi‐π symmetry deriving from a 4d_*xy*_ hybrid (Figure 3 c). The contributions to these bonds are about 10 % U and 90 % Rh character.

**Figure 3:**
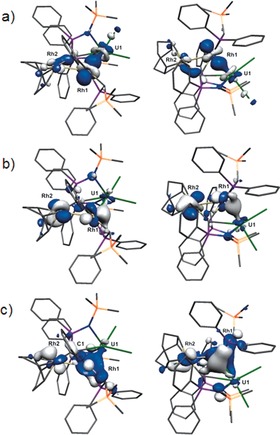
Key Kohn–Sham molecular orbitals representing the principal U−Rh interaction in **2** from two different viewpoints. a) HOMO−7 (352*a*, −5.357 eV), b) HOMO−6 (353*a*, −5.190 eV), c) HOMO−4 (355*a*, −4.923 eV).

To further understand the nature of the U−Rh bond in **2**, we examined the bonding topology of the U−Rh bond with QTAIM.^18^ We find no bond critical point (BCP) between the two rhodium centres, confirming that there is no Rh1−Rh2 bond. A 3,−1 BCP was located for the U1−Rh1 bond, with a local maximum electron density [*ρ*(**r**)] along the bonding path of 0.08, and the electronic energy density term [*H*(**r**)] of −0.04. In comparison to the [(I)UM(μ‐OPAr)_3_] (M=Ni, Pd, Pt) series,5c the U−Rh bond in **2** is slightly higher in electron density at the BCP (0.08 vs. 0.041 to 0.068), indicating stronger orbital interactions, and is more negative for the *H*(**r**) value (−0.04 vs. −0.008 to −0.019). The BCP ellipticity (*ϵ*) provides a benchmark for the symmetry of electron density of a chemical bond:^19^ for a bond of cylindrical distribution of electron density (C−C single (σ) or C≡C triple (σ+2π) bond), the *ϵ* value is approximately 0; for a typical double bond such as C=C bond (σ+π), the *ϵ* value significantly deviates from 0. For the U1−Rh1 bond in **2**, *ϵ* is computed to be 0.24, indicating a double bond interaction. This contrasts to the situation found for [(I)UM(μ‐OPAr)_3_] where *ϵ* values of <0.03 were found, which along with Mayer bond orders of <0.8 and σ‐ and π‐symmetry orbitals being heavily metal‐localised suggested single‐bond character.5c

In conclusion, the combined structural, magnetic, and computational data all consistently suggest that the very short U−Rh bond in **2**, although clearly polarised, is a composite that is equivalent to a net double‐dative bonding interaction where the Rh^I^ donates two electron pairs overall to U^IV^. By the FSR criterion the U−Rh bond in **2** is the shortest isolable actinide–metal bond on record, and by any bond‐order metric the U−Rh bond order is substantially larger than any prior example. This is in contrast to actinide–metal complexes generally, where the majority are weakly single‐bonded and of dominantly σ symmetry,^3^ and may be due to the fact that in **2** Rh1 is coordinated by three anionic ligands and is thus an electron‐rich fragment. Only very recently, a metal–metal multiple dative bond was observed in the gas phase and proposed as a new model for the intermetallic multiple bonds;^8^ complex **2** now adds the first actinide–metal complex prepared in macroscopic quantities and under conventional conditions to feature a novel intermetallic multiple dative bond.^20^

## Conflict of interest

The authors declare no conflict of interest.

## Supporting information

As a service to our authors and readers, this journal provides supporting information supplied by the authors. Such materials are peer reviewed and may be re‐organized for online delivery, but are not copy‐edited or typeset. Technical support issues arising from supporting information (other than missing files) should be addressed to the authors.

Supplementary
