# Supplementary material for: A Very Short Uranium(IV)–Rhodium(I) Bond with Net Double‐Dative Bonding Character
Source: Angew Chem Int Ed Engl. 2018 Apr 27;57(22):6587–91. doi: 10.1002/anie.201803493 (PMC6055764; doi:10.1002/anie.201803493)
Supplement: Supplementary file 1 — Supplementary [file ANIE-57-6587-s001.pdf]

Angewandte Chemie
Eine Zeitschrift der Gesellschaft Deutscher Chemiker

Supporting Information

A Very Short Uranium(IV)–Rhodium(I) Bond with Net Double-Dative Bonding Character

*Erli Lu, Ashley J. Wooles, Matthew Gregson, Philip J. Cobb, and Stephen T. Liddle**

anie_201803493_sm_miscellaneous_information.pdf

Experimental and Computational Details

General Procedures

All manipulations were carried out using Schlenk techniques, or an MBraun UniLab glovebox, under an atmosphere of dry nitrogen. Solvents were dried by passage through activated alumina towers and degassed before use. All solvents were stored over potassium mirrors except for ethers which were stored over activated 4 Å sieves. Deuterated solvent was distilled from potassium, degassed by three freeze-pump-thaw cycles and stored under nitrogen. $[U\{C(PPh_2)(SiMe_3)\}\{C(PPh_2NSiMe_3)_2\}(LiCl)(TMEDA)(\mu\text{-}TMEDA)_{0.5}]_2$ (**1**) was prepared as described previously.[1] Chloro(1,5-cyclooctadiene)rhodium(I) dimer was purchased from Sigma-Aldrich and dried under dynamic vacuum for 12 hours prior to use. ^{1}H and ^{31}P NMR spectra were recorded on a Bruker 400 spectrometer operating at 400.2 and 162.0 MHz respectively; chemical shifts are quoted in ppm and are relative to tetramethyl silane (^{1}H) or external 85% H_3PO_4 (^{31}P). FTIR spectra were recorded on a Bruker Alpha spectrometer with Platinum-ATR module. Variable-temperature magnetic moment data were recorded in an applied dc field of 0.1 T on a Quantum Design MPMS XL7 superconducting quantum interference device (SQUID) magnetometer. Care was taken to ensure complete thermalisation of the sample before each data point was measured and samples were immobilised in an eicosane matrix to prevent sample reorientation during measurements.

Preparation of $[U(Cl)_2\{C(PPh_2NSiMe_3)(PPh[C_6H_4]NSiMe_3)\}\{Rh(COD)\}\{Rh(CH(SiMe_3)\text{-}(PPh_2)\}]$ (2)

At ambient temperature, a solution of $[RhCl(COD)]_2$ (148.6 mg, 0.302 mmol) in toluene (5 ml) was added to a vigorously stirring suspension of **1** (400 mg, 0.151 mmol) in toluene (5 ml). After the addition all the materials was dissolved and a blackish red solution was formed. The mixture was stirred at ambient temperature for 3 days then all volatiles were

removed *in vacuo* to afford blackish red viscous oil. The oil was extracted by 5 ml of toluene and the resultant blackish red solution was concentrated to approximately 1 ml. After standing at −35 °C for 6 days the product **2** was obtained as dark red crystals. Yield: 248.0 mg, 50%. Single crystals suitable for X-ray diffraction were grown from toluene solution at −35 °C. Once obtained as crystalline material, **2** is not soluble in aromatic and aliphatic solvents, and decomposes in coordinative and polar solvents. So, the ^{1}H and ^{31}P NMR spectra of **2** were recorded from the crude product as blackish red viscous oil, along with toluene residue, the cyclooctadiene, and other intractable side products. Therefore, however, this satisfactory ^{13}C and ^{29}Si NMR spectra could not be obtained. The electronic absorption spectrum is also not available for the same reason. Anal. Calcd for $C_{55}H_{69}Cl_2N_2P_3Rh_2Si_3U$, C_7H_8: C 48.29; H 5.03; N 1.82. Found: C 47.96; H 5.25; N 1.40. ^{1}H NMR (C_6D_6, 298 K): δ −27.3 (br, 9 H, -Si*Me*$_3$), −26.9 (s, 3 H), −23.9 (s, 2 H), −23.1 (s, 1 H), −23.0 (br, 9 H, -Si*Me*$_3$), −19.5 (s, 3 H), −16.0 (s, 3 H), −15.7 (s, 1 H), −15.5 (s, 3 H), −12.6 (s, 3 H), −9.2 (s, 4 H), −1.6 (s, 6 H), −0.4 (s, 9 H), 14.4 (s, 1 H), 16.8 (s, 9 H). ^{31}P NMR (C_6D_6, 298 K): δ 17.7 (*d*, $J_{Rh\text{-}P}$ = 91.2 Hz, Rh-(κ^2-C, P-CH($SiMe_3$)*P*Ph_2), −356.1 (s, br), −533.2 (s, br). ATR-IR ν/cm^{-1}: 416 (w), 433 (m), 456 (w), 465 (m), 485 (s), 501 (s), 516 (m), 554 (s), 567 (m), 609 (s), 652 (s), 691 (m), 718 (m), 730 (m), 741 (s), 784 (m), 834 (s), 909 (m), 961 (s), 1015 (m), 1045 (m), 1106 (s), 1245 (s), 1434 (m), 1465 (m), 1978 (w), 2047 (w), 2832 (w), 2877 (w), 2951 (w). Raman ν/cm^{-1} (neat): 76 (s), 192 (w), 415 (m), 442 (s), 456(s), 489 (m), 496(s), 508 (s), 522 (w), 632 (m), 768 (w), 970 (s), 1067 (m), 1109 (w), 1190 (m), 1279 (m), 1406 (w), 1590 (m), 1630 (s), 2808 (m), 3098 (m).

Computational Details

An unrestricted single point energy calculation was performed using coordinates derived from the crystal structure of **2**. No constraints were imposed. The calculation was performed

using the Amsterdam Density Functional (ADF) suite version 2012.01,[2,3] using Slater type orbital (STO) triple-ζ-plus polarisation all-electron basis sets (from the ZORA/TZP database of the ADF suite). Scalar relativistic approaches were used within the ZORA Hamiltonian for the inclusion of relativistic effects and the local density approximation (LDA) with the correlation potential due to Vosko *et al* was used in all of the calculations.[4] Gradient corrections were performed using the functionals of Becke and Perdew.[5,6] MOLEKEL[7] was used to prepare the three-dimensional plots of the electron density. The Atoms in Molecules analysis[8,9] was carried out with Xaim-1.0.[10]

Characterisation Data

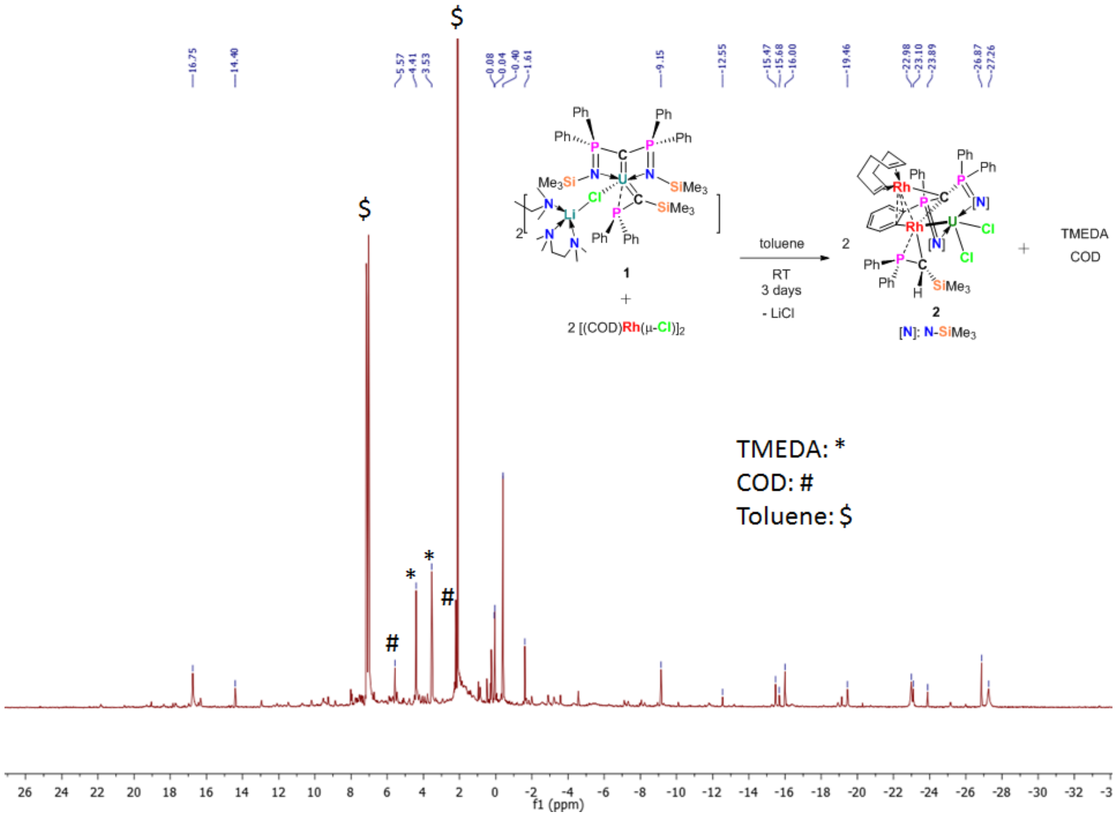

Figure S1. ^{1}H NMR (C_6D_6, 298 K) of the crude product as blackish red viscous oil from reaction between **1** and 2 equivalents of $[RhCl(COD)]_2$.

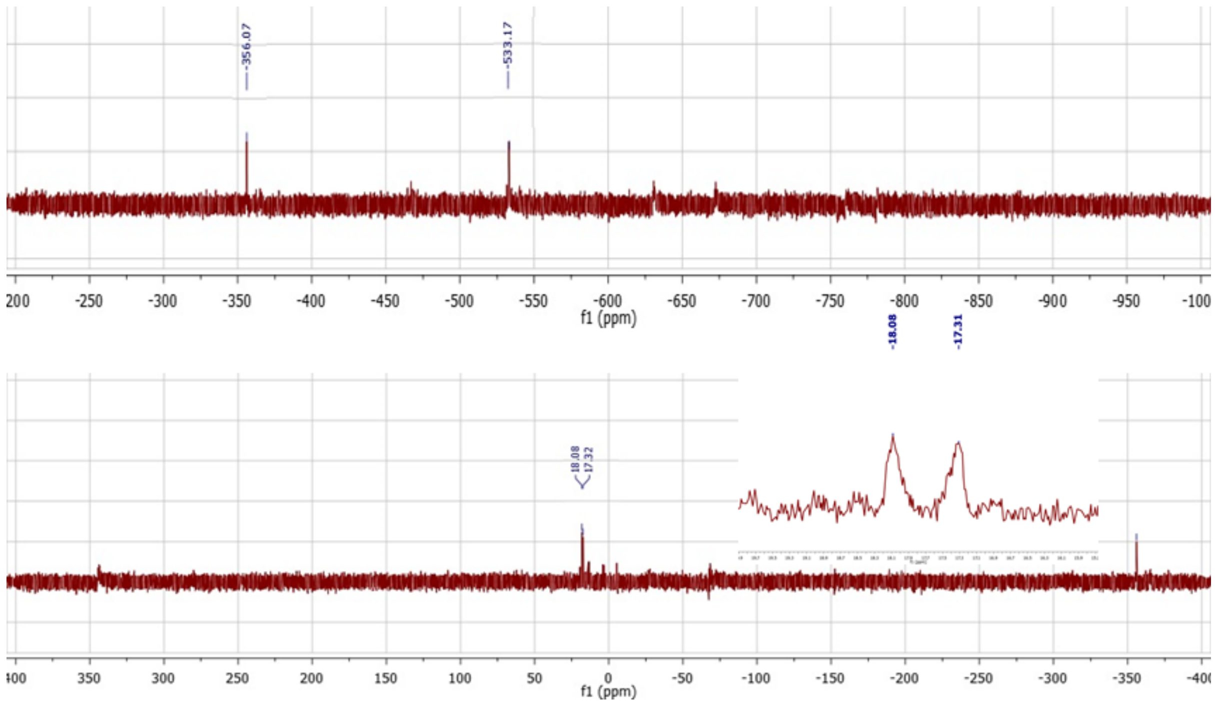

Figure S2. ^{31}P NMR (C_6D_6, 298 K) of the crude product as blackish red viscous oil from reaction between **1** and 2 equivalents of $[RhCl(COD)]_2$. Inset is the enlarged view of the doublet signal at 17.7 ppm.

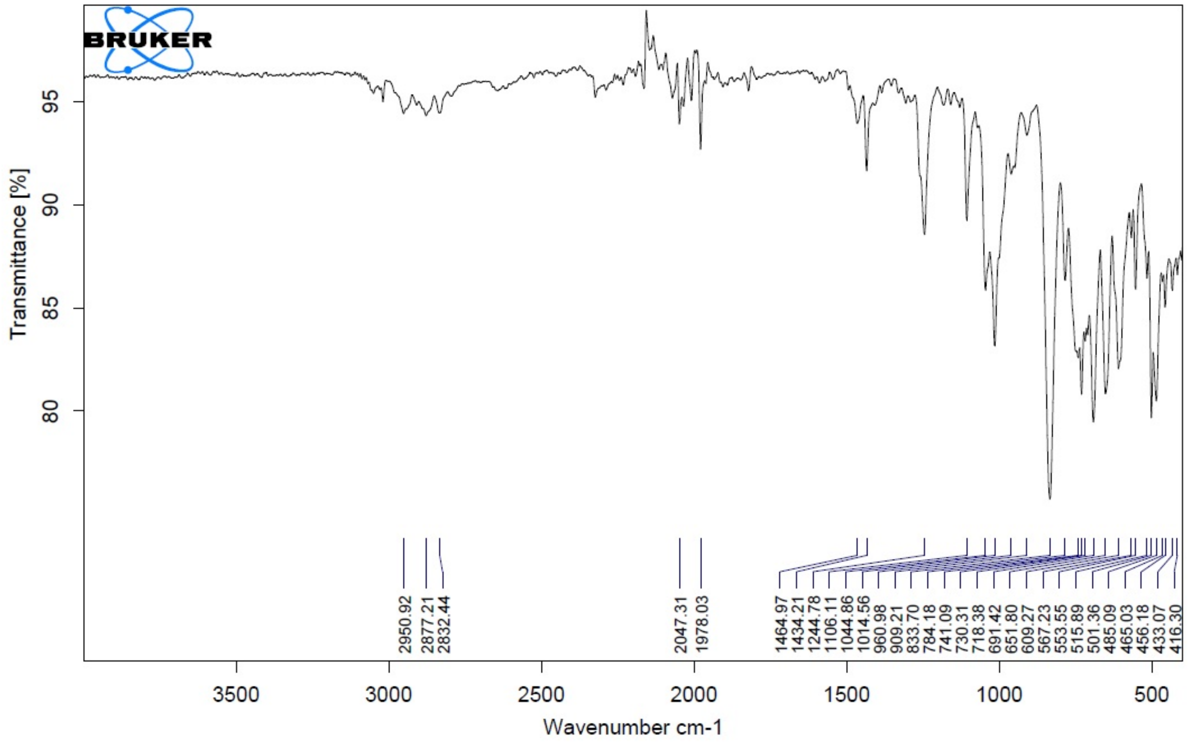

Figure S3. ATR-IR spectrum of **2**.

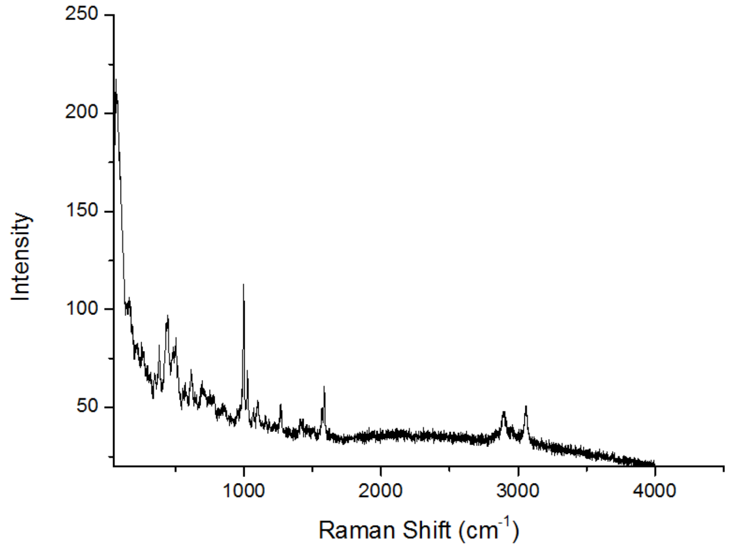

Figure S4. Solid state Raman spectrum of **2**.

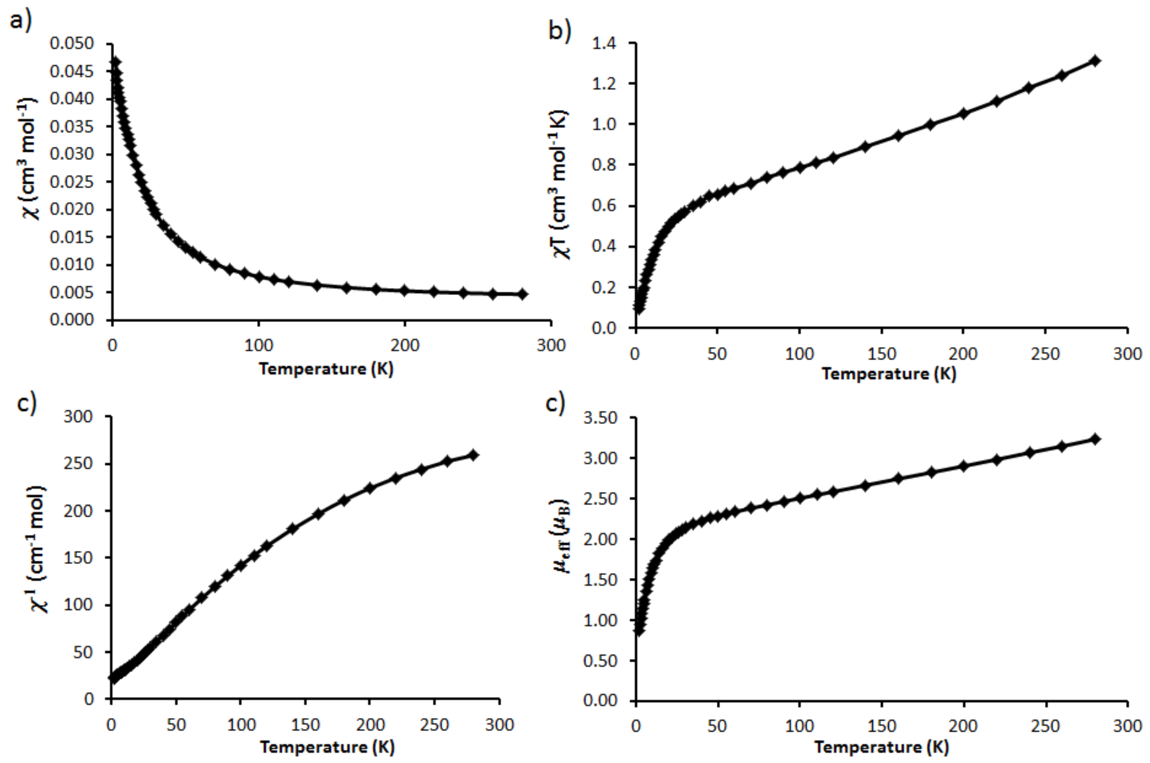

Figure S5. Magnetic data for powdered **2** in a 0.1 T applied magnetic field, presented as: (a) χ vs T; b) χT vs T; c) χ-1 vs T; d) μ_{eff} vs T.

Table S1. Coordinates and Energy of 2 After Single Point Energy Calculation

C	0.24035	-0.22452	-5.80189
C	1.70033	-2.79560	-5.21455
C	3.08861	-0.14521	-4.74432
C	-3.60481	-3.57927	-4.24733
C	-2.27637	-3.92536	-4.14099
C	-2.00548	2.78318	-4.08322
C	-4.04345	-2.36115	-3.77841
C	-1.86728	1.44136	-3.81430
C	-1.36803	-3.04469	-3.55461
C	-1.39421	3.72631	-3.27302
C	-3.15108	-1.47591	-3.19248
C	-1.79019	-1.81456	-3.07475
C	-1.11366	1.02496	-2.68169
C	-0.67912	3.33649	-2.14960
C	-0.49385	1.96911	-1.81622
C	1.97543	5.74410	-1.28316
C	-4.89182	1.66577	-1.36746
C	-0.57759	-5.66192	-1.31122
C	-1.94452	-5.81973	-1.25049
C	-3.58737	2.33565	-0.97581
C	0.01663	-4.50322	-0.80607
C	-2.74052	-4.84033	-0.69483
C	-5.13744	0.35133	-0.61494
C	-0.48690	-0.56892	-0.56851
C	-0.77435	-3.51234	-0.22869
C	-2.16091	-3.69438	-0.17924
C	-3.83641	-0.31543	-0.17453
C	1.89334	3.09289	0.22516
C	-3.17474	2.59286	0.32903
C	3.14315	-4.60393	0.92503
C	-0.06121	5.49935	0.97405
C	4.39775	-1.85310	1.18225
C	-3.20785	-0.09905	1.07694
C	2.92537	5.57965	1.60166
C	-4.03571	2.25059	1.53916
C	4.69531	2.04701	1.94293
C	-3.68601	0.87254	2.12572
C	-0.57754	-1.90992	2.13462
C	5.87061	1.66276	2.59107
C	3.47313	1.56752	2.38829
C	-0.94103	-3.05576	2.83489
C	-0.49248	2.68509	2.86547
C	-0.46441	-0.71139	2.82987
C	0.89229	2.68078	2.96425
C	2.40564	-2.83557	3.24371
C	5.81061	0.80214	3.66585
C	3.43517	0.68824	3.47581
C	-1.27102	3.26945	3.85335

C	4.60291	0.31549	4.11168
C	1.49161	3.28438	4.07812
C	-1.19564	-2.99419	4.20636
C	-0.73287	-0.63465	4.19175
C	-0.67113	3.86994	4.93932
C	-1.09233	-1.78938	4.87509
C	0.69573	3.87774	5.05600
H	0.55449	-0.33154	-6.72402
H	2.26696	-2.80062	-6.01420
H	3.34495	-0.04494	-5.68494
H	-0.63806	-0.64791	-5.70487
H	0.16846	0.72961	-5.58995
H	0.83174	-3.19643	-5.42726
H	-2.52281	3.06468	-4.82859
H	-4.22085	-4.18295	-4.64549
H	-1.97791	-4.76613	-4.46783
H	2.13367	-3.31309	-4.50404
H	-2.27302	0.79644	-4.38173
H	3.01003	0.73985	-4.33096
H	3.77212	-0.66619	-4.27323
H	-4.96052	-2.12652	-3.85663
H	-1.46544	4.64871	-3.48854
H	-0.45292	-3.28947	-3.48347
H	-3.45986	-0.63750	-2.86949
H	-4.88108	1.48323	-2.34036
H	1.18288	5.66090	-1.85363
H	-0.03828	-6.34223	-1.69699
H	-2.34269	-6.61049	-1.59488
H	2.73557	5.29945	-1.71298
H	-3.26809	3.01233	-1.63911
H	-0.30278	4.00364	-1.58774
H	2.18414	6.69247	-1.15043
H	-5.64078	2.28680	-1.18463
H	-5.63845	-0.26955	-1.20121
H	0.95857	-4.39087	-0.85635
H	-3.68350	-4.95210	-0.66587
H	-3.74018	-1.25325	-0.50823
H	2.77645	2.97104	-0.22792
H	3.33879	-4.64553	-0.03435
H	-5.69546	0.53311	0.18239
H	-2.70966	-3.02480	0.21194
H	4.82761	-2.11999	0.34294
H	-0.75426	5.07477	0.42662
H	-2.61952	3.41789	0.43406
H	-0.12964	6.47322	0.89045
H	2.35284	-5.14933	1.12114
H	4.73287	2.63584	1.19840
H	4.18611	-0.89675	1.15117
H	-4.98924	2.25877	1.27292
H	3.02908	6.54746	1.48673

H	3.78332	5.13696	1.43300
H	3.90996	-4.94655	1.42996
H	-2.76613	-0.91358	1.45280
H	-0.18267	5.24250	1.91199
H	5.00542	-2.02897	1.93073
H	6.70947	1.99364	2.29210
H	-3.90840	2.94272	2.23550
H	-0.91118	2.28342	2.11330
H	2.63372	5.38879	2.51759
H	-1.01627	-3.88495	2.37739
H	-0.19774	0.07251	2.36412
H	-4.48644	0.49849	2.57245
H	-2.97992	0.98238	2.81104
H	1.69783	-3.48980	3.42037
H	2.09937	-1.94329	3.50894
H	3.20544	-3.07277	3.75805
H	-2.21819	3.25599	3.78146
H	6.61233	0.54172	4.10425
H	2.60215	0.34620	3.77827
H	2.43752	3.28898	4.16614
H	-1.44009	-3.78066	4.67983
H	-0.67138	0.19604	4.64866
H	4.57259	-0.27534	4.85499
H	-1.20575	4.27972	5.60913
H	1.10255	4.29040	5.80875
H	-1.26832	-1.75042	5.80781
Cl	3.77075	-2.45363	-2.14710
Cl	4.39067	1.14194	-1.43469
N	0.89433	-0.93369	-2.97625
N	1.60131	-2.07626	0.40873
P	-0.58248	-0.66674	-2.33932
P	-0.01857	-1.99096	0.40529
P	1.88024	1.97758	1.59063
Rh	-2.08699	0.85544	-0.44659
Rh	0.70879	1.31307	-0.25016
Si	1.44917	-1.02956	-4.63729
Si	1.63636	4.93666	0.38432
Si	2.81556	-2.83735	1.41605
U	2.31616	-0.48369	-1.17875

Energy: −789.96217456 eV

References

1. E. Lu, J. T. Boronski, M. Gregson, A. J. Wooles, S. T. Liddle, *Angew. Chem. Int. Ed.* **2018**, 10.1002/anie.201802080.
2. C. Fonseca Guerra, J. G. Snijders, G. Te Velde, E. J. Baerends, *Theor. Chem. Acc.* **1998**, *99*, 391.
3. G. Te Velde, F. M. Bickelhaupt, S. J. van Gisbergen, A. C. Fonseca Guerra, E. J. Baerends, J. G. Snijders, T. Ziegler, *J. Comput. Chem.* **2001**, *22*, 931.
4. S. H. Vosko, L. Wilk, M. Nusair, *Can. J. Phys.* **1980**, *58*, 1200.
5. A. D. Becke, *Phys. Rev. A.* **1988**, *38*, 3098.
6. J. P. Perdew, *Phys. Rev. B.* **1986**, *33*, 8822.
7. S. Portmann, H. P. Luthi, *Chimia* **2000**, *54*, 766.
8. R. F. W. Bader, *Atoms in Molecules: A Quantum Theory*, Oxford University Press, New York, 1990.
9. R. F. W. Bader, *J. Phys. Chem. A* **1998**, *102*, 7314.
10. http://www.quimica.urv.es/XAIM.
